# Barriers and facilitators of access to sexual and reproductive health services among migrant, internally displaced, asylum seeking and refugee women: A scoping review

**DOI:** 10.1371/journal.pone.0291486

**Published:** 2023-09-14

**Authors:** Pengdewendé Maurice Sawadogo, Drissa Sia, Yentéma Onadja, Idrissa Beogo, Gabriel Sangli, Nathalie Sawadogo, Assé Gnambani, Gaëtan Bassinga, Stephanie Robins, Eric Tchouaket Nguemeleu

**Affiliations:** 1 Institut Supérieur des Sciences de la Population, Université Joseph Ki-Zerbo, (ISSP/UJKZ), Ouagadougou, Burkina Faso; 2 Département Sciences Infirmières, Université du Québec en Outaouais, Saint-Jerôme, Québec, Canada; 3 École des Sciences Infirmières, School of Nursing, Faculty of Health Sciences, University of Ottawa, Ottawa, Ontario, Canada; University of Greenwich, UNITED KINGDOM

## Abstract

**Introduction:**

Migrant, internally displaced, asylum seeking and refugee women experience ongoing risks of having their reproductive healthcare rights violated. This ever-increasing population also has limited access to sexual and reproductive health services. We conducted a scoping review to identify the barriers and facilitating factors when accessing sexual and reproductive health services for this specific population.

**Methods:**

We searched the grey literature and queried eight bibliographic databases (Embase, Medline, Cinahl, Scopus, Science Direct, Web of Science, Hinari, and Cochrane Library) to extract articles published between January, 2000, and October, 2021. The extracted data were organized in a framework adapted from Peters et al. and then categorized as facilitators or barriers. We followed the Arksey and O’Malley framework and wrote the report according to the PRISMA-Scr recommendations.

**Results:**

The search identified 4,722 records of which forty-two (42) met eligibility criteria and were retained for analysis. Ten (10) groups of factors facilitating and/or limiting access to sexual and reproductive health care emerged from the synthesis of the retained articles. The main barriers were lack of knowledge about services, cultural unacceptability of services, financial inaccessibility, and language barriers between patients and healthcare providers. Facilitators included mobile applications for translation and telehealth consultations, patients having a wide availability of information sources, the availability health promotion representatives, and healthcare providers being trained in cultural sensitivity, communication and person-centered care.

**Conclusion:**

Ensuring the sexual and reproductive rights of migrant, internally displaced, asylum-seeking and refugee women requires that policymakers and health authorities develop intervention strategies based on barriers and facilitators identified in this scoping review. Therefore, considering their mental health in future studies would enable a better understanding of the barriers and facilitators of access to sexual and reproductive health services.

## Introduction

Migrant, internally displaced, asylum seeking and refugee women represent a vulnerable group whose number is constantly growing. According to the Office of the United Nations High Commissioner for Refugees (UNHCR), the number of migrants, internally displaced persons (IDPs), asylum seekers and refugees worldwide reached 82 million in 2020, a 28 per cent increase from 2015 [1]. These women live in precarious conditions that increase the probability of their reproductive healthcare rights will be violated. For example, both women and adolescent girls living in internally displaced persons (IDPs) and refugees camps have increased risk of contracting sexually transmitted infections, having unwanted pregnancies and abortions [2, 3]. Despite these increased risks, healthcare centers in host localities do not always take these concerns into account, resulting in women having limited access to appropriate sexual and reproductive health (SRH) services [4, 5]. SRH services include prenatal care, childbirth care, newborn care, family planning, safe abortion, and the management of sexually transmitted infections (STI) and human immunodeficiency virus (HIV) [6].

Access to SRH services is a fundamental human right that was highlighted at the 1994 International Conference on Population and Development (ICPD) and reinforced in the priorities set out in the 2030 Agenda of the Sustainable Development Goals [7]. In these commitments to healthcare, legislators and public healthcare authorities have been mandated to ensure that all individuals, without discrimination, have universal access to SRH services. Given the vulnerability of migrants, IDPs, asylum seekers and refugees, specific evidence-based measures are needed to promote their access to SRH services. For this purpose, it is important to identify barriers and facilitators of access to SRH care for migrant, internally displaced, asylum seeking and refugee women. The studies that have examined this issue provided insights into the influence of communication and socio-cultural factors as well as factors related to the quality of services that facilitate or limit access to SRH services for migrant, internally displaced, asylum seeking and refugee women. Two review articles on barriers and facilitators of access to SRH care specifically focused on adolescent girls and young women [2, 8], while another concerned adult women aged 18 to 64 years but was limited to preventive SRH care, excluding maternity care, obstetric care and HIV/STI prevention [9]. Therefore, much remains unknown about barriers and facilitators of access to other relevant SRH services (including prenatal care, childbirth, postnatal care, HIV/STI) for migrant, internally displaced, asylum seeking and refugee women.

This study aims to provide evidence-based data that may serve to improve the access to and use of SRH services for migrant, internally displaced, asylum seeking and refugee women. This review concerns women of all age, from early adolescents to older adults. It answers the following question: according to the scientific literature, what are the barriers and facilitators of access of sexual and reproductive health care for migrant, internally displaced, asylum seeking and refugee women?

## Methods

This study is a scoping review of the scientific literature based on the framework of Arksey & O’Malley [10]. The findings are reported as per the recommendations of the Preferred Reporting Items for Systematic Reviews and Meta-Analyses extension for Scoping Reviews (PRISMA-Scr) [11]. The reviews were registered within Research Registry (https://www.researchregistry.com/register-now#registryofsystematicreviewsmeta-analyses/, reviewregistry1394).

### Eligibility criteria

Inclusion criteria were based on the population to be included, the risk factors to be considered, the design of the studies, the geographic scope, and the timeframe. For inclusion, selected articles must:

1. Be published in French or English, the working languages of the research team;
2. Be published between January 1^st^, 2000 and October 15^th^, 2021. The year 2000 was the deadline to ensure universal access to healthcare [12], including for migrants, internally displaced persons, asylum seekers or refugees. We thus considered this year as the starting point of our study period;
3. Include data on females who were 12 years old or older. We considered this age group because an earlier study showed that some girls are sexually active by the age of 12 [13];
4. Describe migrant, internally displaced, asylum seeking or refugee women;
5. Focus on access to SRH, including prenatal consultations, childbirth, postnatal care, immunization, healthy infant care, family planning, and management of sexually transmitted infections;
6. Focus on barriers and facilitators;
7. Editorials, commentaries, methodological guides, manuals, and review articles (including systematic reviews) were excluded.

### Data sources and search strategy

We developed a search strategy using keywords based on the eligibility criteria. The keywords used included both free and controlled vocabulary. These keywords refer to the study population (refugee or "asylum seeker" or displaced or migrant), the type of services (“healthcare accessibility”) and the focus of the study (barriers or obstacles or “facilitating factors”). Spelling variants and synonyms of the keywords were also considered in the construction of the search syntax. We queried eight databases: Embase, Medline, Cinahl, Scopus, Science Direct, Web of Science, Hinari, and Cochrane Library. We also searched grey literature on Open Grey database, on the United Nations Office for the Coordination of Humanitarian Affairs (OCHA) website and on the UNCHR website. The complete search syntax applied in each database is presented in S1 Appendix.

### Selection of articles

**Fig 1:**
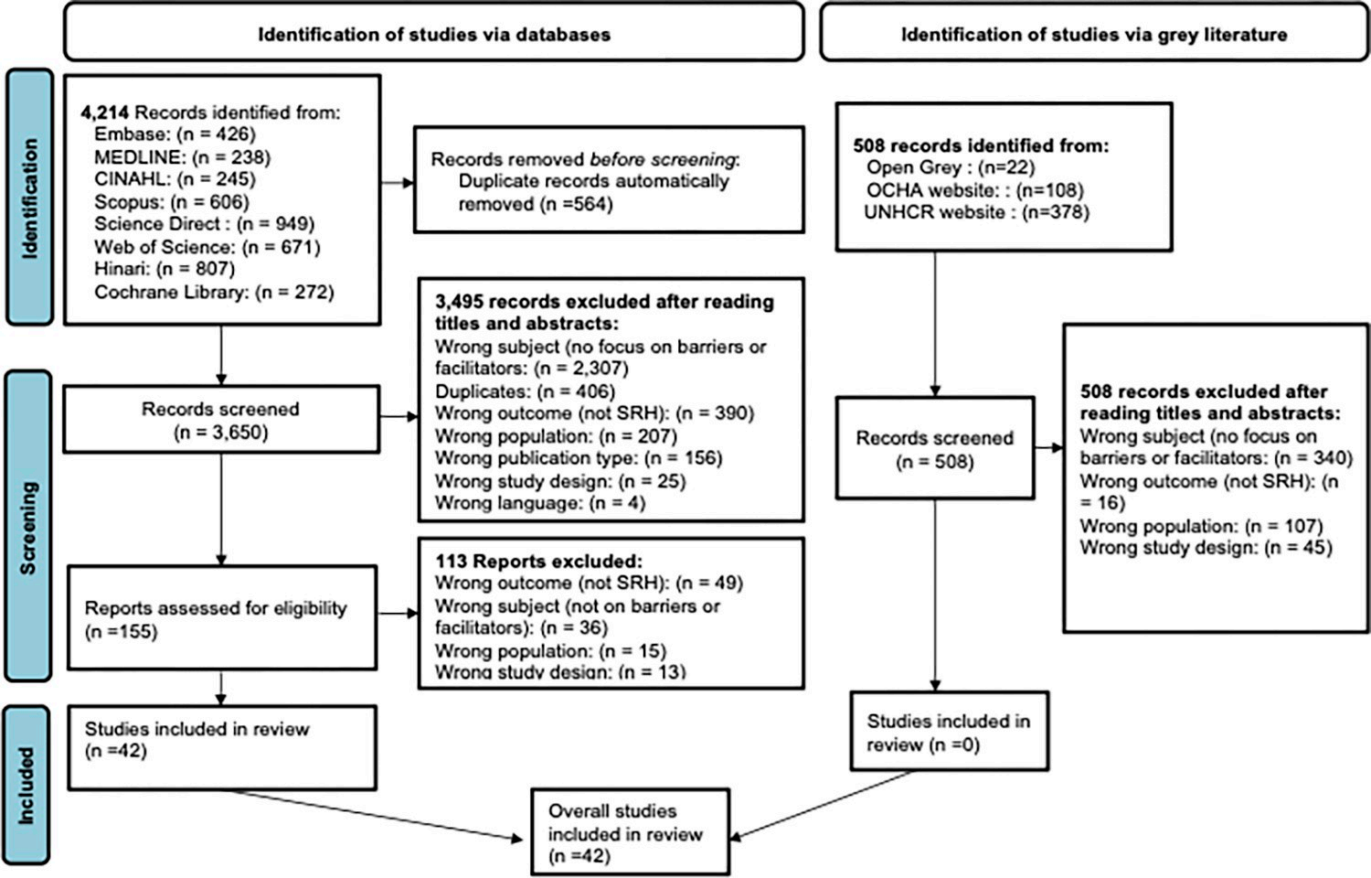
Prisma flow-chart of identified and screened records.

The selection of articles was done in several steps. In the first step, the search strategies were applied to the databases to retrieve the references of the articles whose title, abstract or keywords contained the words composing the search equations. Subsequently, the records retrieved were imported into an EndNote library where duplicates were detected and removed. Articles were then imported into Rayyan for the selection of articles according to eligibility criteria. Authors PMS, SD and ETN reviewed the titles and abstracts according to the process described in Fig 1. In the case of disagreement, authors YO and SG independently reviewed titles and abstracts and their decision was used to resolve the conflict. The full texts of the selected articles were then uploaded into Rayyan and read by four co-investigators (SPM, SG, NS and AG) to eliminate those that did not meet eligibility criteria. In the event of a discrepancy, a co-investigator (IB or SR) reviewed the given article to settle the conflict.

### Data extraction

Articles were organized according to the geographical area where the study was conducted. Then, using a seven-item data extraction grid, we extracted the data. The seven items included: references, country of study, study population, type of study, type of service, facilitating factors and barriers. To ensure that the extraction grid was understood and used in a uniform manner, data extracted from the first five articles was done as a team during two working meetings. During this group extraction phase, no amendments were made to the form. Subsequently, the data from the remaining articles were extracted by authors PMS SD, MG and ETN.

### Data analysis

The extracted factors were organized according to Peters et al. framework and adapted for an analysis of access to healthcare in the context of instability [14]. The framework centers on four main factors of access, notably: availability, geographical accessibility, financial accessibility, and acceptability. In addition, our review identified six other relevant factors, including: patient-provider communication, client knowledge, decision-making autonomy, stigma/discrimination, and administrative factors. Each factor was considered in the model and categorized as a facilitator or barrier. Finally, the frequency of the identified factors is reported.

## Results

Fig 1 shows the record selection process. A total of 4,124 records were identified from the eight bibliographic databases. Upon importing the references into EndNote software, 564 duplicates were automatically deleted, leaving 3,650 records. The remaining references were imported into the Rayyan platform. A review of these titles and abstracts resulted in the selection of 155 references whose full texts were reviewed. In the end, 42 articles were retained for data extraction. In addition, 508 grey literature references were identified. However, none met the eligibility criteria.

### Characteristics of the retained articles

The characteristics of the 42 selected articles are presented in Table 1. The studies spanned 30 countries. The Asian continent was the most represented with 16 articles describing studies in 12 countries, followed by the African continent (13 studies) which were mainly about Uganda (n = 5), Kenya (n = 3) and Ghana (n = 3). Methodologically, 29 studies were qualitative, ten studies used a mixed methods approach, and three were quantitative. In addition, 15 of the 42 studies focused on SRH in general and described "reproductive healthcare services" or "maternity services”. The remaining 27 studies focused on one or more specific services, including primarily prenatal healthcare, contraceptive/family planning, and childbirth services.

**Table 1:** Characteristics of retained articles.

	References	Location	Study population	Type of study	Types of service	Facilitating factors	Barriers
	Sub Saharans living in African countries						
1.	Arnold et al, 2014 [21]	Nairobi (Kenya)	Government authorities; healthcare providers; Immigrant and aboriginal women	Qualitative	General care with SRH (maternity) services taken into account		Threat of harassment; cost differentials between migrant and Kenyan women; real or perceived discrimination; requirement for documentation and language barriers
2.	Yiran et al, 2015 [22]	Accra (Ghana)	Immigrant women; street vendors	Mixed methods	Maternal healthcare services		Lack of healthcare facilities; low income; high cost of maternal healthcare despite being free; long waiting lists and the belief that traditional medicines are sufficient to protect pregnant women and their babies
3.	Baada et al, 2021 [23]	Ghana	Immigrant women	Qualitative (individual and focus group interviews)	Family planning, childbirth, prenatal care		Low autonomy in family planning decision making or reproductive choices: having children; poor perception of quality of healthcare services; financial barriers; professional occupations; distance from healthcare centers
4.	Nara, Banura and Foster, 2020 [24]	Refugee camps (Uganda)	Congolese refugee women in urban camps.	Qualitative	Emergency contraception		Medication shortages; high cost of services; lack of knowledge of contraceptive methods; use of other medications (anti-malarial drugs, analgesics)
5.	Zepro and Ahmed, 2016 [25]	Ethiopia	Internally displaced women	Mixed methods	Assisted childbirth	Apparent good health; experience with childbirth Partner’s level of education assistance of relatives during home deliveries	Apparent good health; experience with childbirth; lack of knowledge; partner’s decision; partner’s low level of education; long waits; low quality services; distance to birthing centers; cultural and religious beliefs; assistance of relatives during home deliveries
6.	Deker and Constantine, 2011 [26]	Angola	Internally displaced women; healthcare providers	Mixed methods	Use of contraceptive methods		Poverty; difficulty paying for services; distance to services; limited knowledge of contraceptive methods
7.	Tanabe et al, 2017 [27]	Bangladesh, Djibouti, Kenya, Malaysia, and Uganda	Refugee women	Mixed methods	Use of contraceptive methods	Presence of interpreters; information on family planning during home visits	Remoteness of services; cost of transportation; lack of knowledge about contraceptive methods; religious beliefs; stigma; language barriers; discrimination; disapproval of sex among adolescents; high healthcare provider workload
8.	Tanabe M et al, 2015 [28]	Uganda, Kenya, and Nepal	Refugee women living with a physical, sensory, intellectual, or mental disability (aged 20–49); Men	Qualitative	Family planning and other SRH services.		Lack of knowledge about SRH; discrimination; lack of respect from healthcare providers; social rejection of extra-marital pregnancies
9.	Orach et al, 2009 [29]	Uganda	Internally displaced women	Mixed methods	Childbirth center		Lack of financial resources; lack of information; low decision-making power of women
10.	Munemo et al, 2020 [15]	Ghana	Immigrant women; street vendors; key informants	Qualitative	Family planning	Support from partners	Lack of consent from partners (including physical violence, divorce); misinformation about medication side effects (accusations of prostitution against those using family planning)
11.	Mwenyango, 2020 [16]	Uganda	Refugee women; key informants	Mixed methods	Family planning		Communication problems (language barriers, lack of knowledge about available services); lack of human and material resources (specialized care, staff, equipment, medication,); long wait times; lack of courtesy and respect from healthcare service providers; lack of coordination between service providers in the camps; poverty of refugees; low autonomy of women.
12.	Seyife, 2019 [30]	Ethiopia	Refugee women aged 15–49	Quantitative	Family planning		Older age; no spouse, lack of employment; poor location of healthcare service delivery site; low decision-making power of women
13.	Munyaneza et al, 2019 [31]	South Africa	Refugee women aged 18–49 who used public services	Qualitative	Reproductive healthcare services	Quality care offered; social support	Xenophobia of providers; discrimination; feeling unwelcome; lack of professionalism (issues of confidentiality, abuse); language barriers; fear; insufficient healthcare personnel
Sub Saharans living elsewhere							
14.	Ahrne et al, 2019 [32]	Sweden	Immigrant women of Somali origin; healthcare providers	Qualitative (focus group)	Prenatal care	Community group care provision (privacy and stigma challenges); provision of person- centered care	Stereotypes; language barriers
15.	Gele A A et al, 2020 [33]	Norway	Somali immigrant women living in Oslo	Qualitative (individual interviews)	Use of contraceptive methods	Communication in native language; enhanced multicultural communication skills among caregivers; partnership with community leaders; women’s empowerment	Language barriers; high cost of contraceptive methods; lack of appropriate information; religious beliefs; pro-natalist social culture; partner opposition
16.	Van den Bos and Sabar, 2019 [34]	Israel	Eritrean refugee women residing in Israel	Mixed methods (individual interviews)	Prenatal care		Lack of permission from employers to visit the healthcare center; language barriers
17.	Mehta P. K. et al, 2018 [17]	Boston (USA)	Congolese and Somali refugee and immigrant women residing in Boston	Qualitative (group interviews)	Gynaecological care		Stigma; unmarried status, cultural discomfort of being examined naked; lack of partner support (permission to go, jealousy); insufficient resources to pay for care (insurance)
Elsewhere in the world							
18.	Nabieva et al, 2019 [35]	Isfara (Tajikistan)	Immigrant women, mothers-in-law, healthcare providers	Qualitative (individual and group interviews)	Prenatal care; childbirth		Delayed decision making: low maternal autonomy; influence of mothers in law; cohabitation with grandparents; role allocation; beliefs about pregnancy and childbirth; myths about health services
19.	Ceulemans et al, 2020 [36]	Belgium	Arabic- speaking pregnant women	Qualitative (individual interviews)	Prenatal consultation	Presence of interpreters	Language barriers; preference for natural remedies
20.	Bitar et al, 2020 [37]	Sweden	Arabic- speaking pregnant women	Qualitative (individual interviews)	Prenatal care	Use of a mobile phone application to communicate with women	
21.	Schmidt et al, 2018 [38]	Switzerland (Geneva)	Immigrant women 18 years and older	Qualitative (focus group)	Reproductive healthcare services	Provision of simple communication materials in several languages; multicultural training for healthcare providers; provision of specially trained nurses or social workers to guide migrants through the health system	Financial accessibility; language barriers; discrimination (real or perceived); lack of information; embarrassment
22.	Tobin et al, 2014 [19]	Ireland	Women asylum seekers	Qualitative (individual interview)	Childbirth		Insufficient adaptation of maternity services to meet needs; healthcare providers lack multicultural training; limited access to interpreters
23.	Lee et al, 2014 [39]	Canada (Toronto)	Immigrant women of Chinese origin	Qualitative (individual interview)	Maternity services	Multicultural and multilingual training for healthcare providers; diversity of sources of information about pregnancy and childbirth	Limitations in the choice of providers to deliver care
24.	Betancourt et al, 2013 [40]	USA (New York)	Immigrant women of Mexican origin	Quantitative and qualitative (focus group)	Reproductive healthcare services	Access to translation service; access to a health promotion officer (“promotora”)	Lack of knowledge; cost of services; language barriers
25.	Su et al, 2014 [41]	China (Chong Qing)	Immigrant women working in a business	Qualitative (individual and group interviews)	Reproductive healthcare services		Lack of knowledge; high cost of care; long waiting time; supply not adapted to needs; mistrust concerning lack of confidentiality
26.	Kim et al, 2012 [42]	Vietnam	Immigrant women working in a business	Mixed methods including individual and group interviews	Management of sexually transmitted infections		Social representations (unmarried women should not have sex nor receive gynecological care); fear of pay cuts due to absence from work to attend healthcare centers; lack of information; high cost of services
27.	Metusela et al, 2017 [18]	Australia and Canada	Immigrant and refugee women	Qualitative (individual and group interviews)	Reproductive healthcare services: Human Papilloma Virus (HPV) vaccination, cervical cancer screening, contraception		Lack of knowledge about the menstrual cycle; discussions of sexuality being socially unacceptable; social representation of cervical cancer screening and human papilloma virus (HPV) vaccination as incompatible with the requirements for virginity; pro-natalist traditions; prejudice about family planning (thought of as ineffective or as a form of abortion)
28.	Dadras et al, 2020 [43]	Iran	Pregnant immigrant women	Qualitative (individual interviews)	Prenatal services		Financial constraints; unaffordable health insurance; feeling discriminated against (e.g., being asked about nationality, the tone of voice); stigma; long waits; lack of decision- making autonomy; lack of female healthcare providers in maternity services; illegal migration status (visa expiration)
29.	Nellums et al, 2021 [44]	England	Undocumented immigrant women	Qualitative (individual interviews)	Maternity services (prenatal care and childbirth)		Financial barriers; illegal migration status
30.	Siddaiah et al, 2018 [45]	India	Immigrant women aged 15–49	Mixed methods including individual and group interviews	Services (prenatal care and childbirth)	Reproductive health awareness; conducting home visits; deploying mobile strategies to reach migrant women in their workplaces	Lack of financial resources; disruptions in continuity of healthcare service availability; lack of knowledge about prenatal care and childbirth; misconceptions and mistrust of the public health system; lack of transportation
31.	Pardhi et al, 2020 [46]	India	Internally displaced pregnant women and mothers of children under the age of two	Qualitative (individual interviews)	Prenatal care and vaccination		Perception of lower importance of prenatal care in relation to their IDPs status; language barriers; lack of awareness of healthcare centers’ location(s)
32.	Habersack et al., 2011 [47]	Australia	Immigrant women, and healthcare service providers (public or NGO)	Qualitative (individual interview)	Prenatal care	Mobile outreach service; collaboration with community leaders; training of healthcare staff in respecting cultural differences; cultural and religious neutrality of health services; dissemination of message in native? language; use of professional translators	Lack of communication about the availability of services; language barriers; inappropriate infrastructure (lack of visual and auditory privacy); presence of religious symbols that are not culturally appropriate; lack of cultural competence and cultural insensitivity
33.	Funge et al, 2020 [48]	Denmark	Women who are pregnant or have given birth in the last two months; undocumented immigrants	Qualitative (individual interviews)	Prenatal care	Support of relatives for translation and accompanying women to healthcare center	Fear of deportation; financial barriers; lack of knowledge of procedures for accessing services; distance from health centers; lack of continuity of services
34.	Lin et al, 2018 [49]	China	Immigrant women who have recently attended received services; Healthcare providers	Qualitative (individual interview, group interview)	Prenatal care	Use of a phone platform (WeChat) to disseminate information	Lack of knowledge about prenatal care; stigma; discrimination; communication failures
35.	Talhouk et al, 2016 [50]	Lebanon	Syrian refugee women	Qualitative (individual interview)	Prenatal care	Use of a mobile phone application to raise awareness	
36.	Kaneoka et al. 2019 [20]	England	Refugee and internally displaced women	Qualitative (individual interview)	Reproductive health information	Development of information tools in several languages	Sexual and reproductive health information unavailable; language barriers; cultural and religious values (pro-natalist, being examined by male healthcare providers, prohibition of sex outside of marriage); difference in sources of SRH information between their home and host countries
37.	Dickmen et al, 2019 [51]	Turkey	Syrian immigrant women	Quantitative	Family planning services	Support from partners	Pro-natalist cultural and religious values; low income; low education of partner; lack of social security
38.	Fahme et al, 2021 [52]	Lebanon	SRH care providers for Syrian adolescent refugees; Educators	Qualitative		Involvement of men and parents in reproductive health communication; multidimensional approach in the development of any SRH intervention for adolescents: cultural norms, empowerment, peer education	Insufficient knowledge of reproductive health among adolescent girls, low autonomy of adolescent girls; insufficient communication of reproductive health among parents; stigmatization of premarital sex; low involvement of men (e.g., not accompanying women to the health center)
39.	Khin et al, 2021 [53]	Japan (Tokyo)	Immigrant women	Qualitative	Contraception	Use of an interpreter at the health center	Language barriers (posters in a language that is not accessible, in communicating with caregivers); lack of information sources; beliefs (side effects of contraceptive methods, fear of loss of fertility); taboo about discussing sexuality, woman’s body should only be seen by her spouse; financial inaccessibility: high cost of contraceptives
40.	Makuch et al, 2021 [54]	Brazil	Immigrant women of Venezuelan origin	Qualitative (group interviews),	Prenatal care, childbirth, contraception		Language barriers; discrimination in the offer of services based on the belief that migrants usurp services reserved for native citizens; difficulties in accessing the first prenatal visit; long wait times in health centers; lack of transportation for women in labor; prohibition of companionship for women in labor as is done in their country of origin; lack of supply of a full range of contraceptives
41.	Bains et al, 2021 [55]	Norway	Pregnant immigrant women; Immigrants who have given birth; midwives	Mixed Methods with Interviews and questionnaire	Prenatal care, childbirth		Lack of knowledge of the organization of the health care system/available services; long waiting time for consultations; language barriers including lack of an interpreter, respect for anonymity and confidentiality with the presence of an interpreter; structural barriers (access to transportation, financial reasons, obtaining a leave of absence from work to get care); dissatisfaction with expectations (e.g. need to carry out ritual practices before and after childbirth such as ear piercing and taking a bath)
42.	Korri et al, 2021 [56]	Lebanon	Refugee women	Quantitative	Sexually transmitted infection care, Prenatal care Family planning		Lack of knowledge about reproductive health services; feeling mistreated by staff; high cost of care; long wait times; long distance to health facility

Women receiving healthcare services were interviewed in all 42 studies. In addition, healthcare providers were interviewed in seven of the 42 studies. Two studies included key informants [15, 16]. With respect to the profile of the study population, 24 of 42 studies focused exclusively on immigrant women, ten on refugee women, and four on internally displaced persons. Two studies included both refugees and immigrants [17, 18], one study included asylum seekers [19], and one study included refugees and internally displaced persons [20].

### Barriers and facilitators of access to sexual and reproductive health services

**Fig 2:**
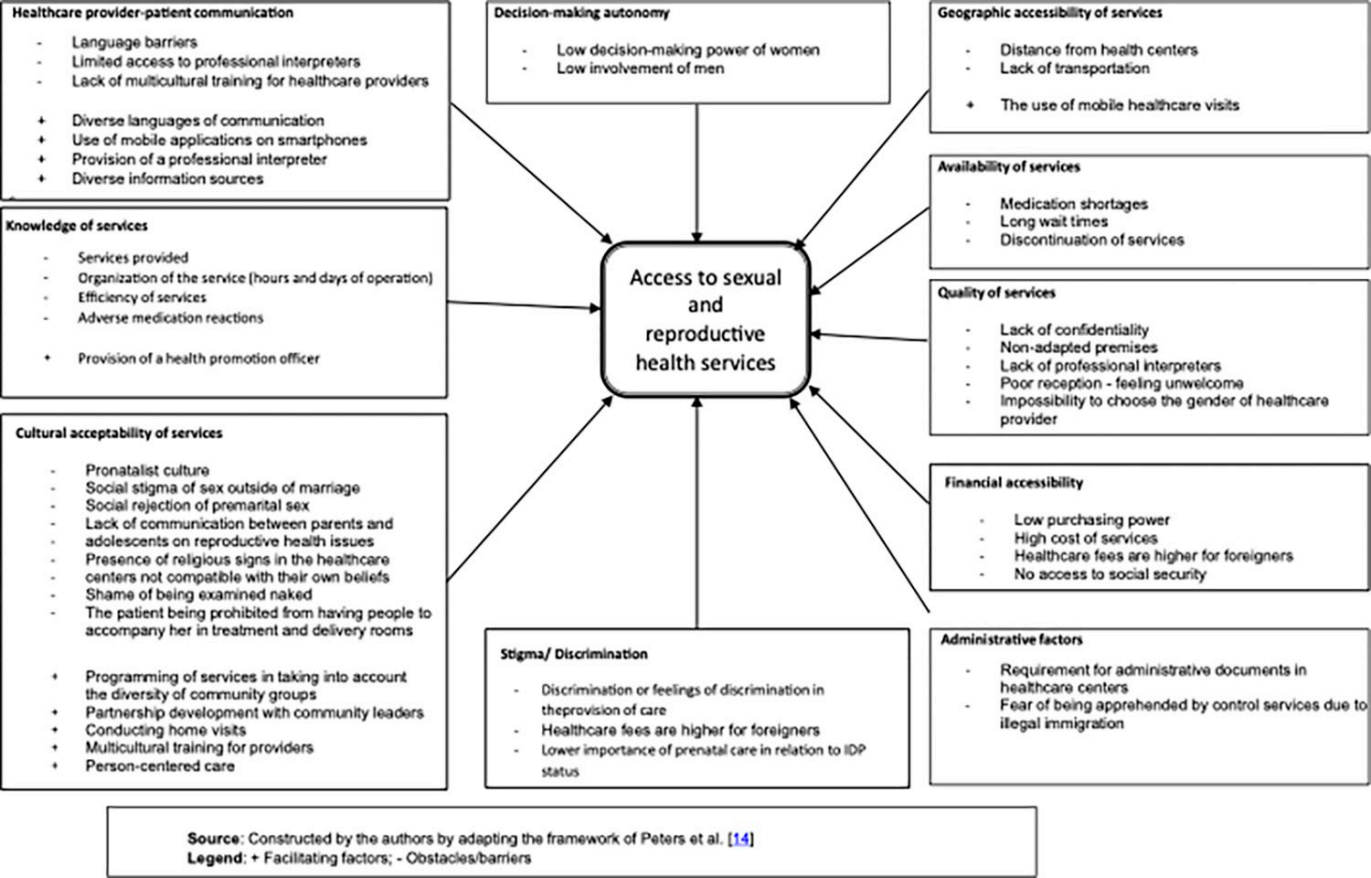
Conceptual framework of facilitating factors and barriers to access to reproductive health services by migrant, internally displaced, asylum seeking and refugee women.

Fig 2 presents all the factors identified as facilitating or constraining the use of SRH by migrant, internally displaced, asylum seeking and refugee women. Factors were grouped into ten groups: geographic accessibility of services, availability of services, quality of services, communication, financial accessibility of services, knowledge of services by beneficiaries, cultural accessibility of services, stigma/discrimination, women’s decision-making autonomy and administrative factors.

### Geographic accessibility of services

Six of the 42 studies described geographic accessibility to healthcare services that impede access to SRH services for migrant, internally displaced, asylum seeking and refugee women. These include the distance from healthcare centers [22, 27, 48, 56] and lack of transportation [27, 45, 54]. On the other hand, two studies reported that the use of mobile healthcare teams in migrant welcome sites as a factor that improved the use of SRH services [45, 47].

### Availability of services

Nine of the 42 studies reported barriers related to the availability of SRH services that handicapped their use. These included lack of supply of medications [24], long wait times for care [16, 22, 41, 43, 54, 56] and unavailability of services at certain hours of the day or days of the week [16, 45, 48, 54].

### Quality of services

Eleven of 42 studies described barriers inherent in the quality of SRH services that were barriers to their use by migrant, internally displaced, asylum seeking and refugee women. Five articles noted a lack of confidentiality when receiving care [30, 31, 36, 41, 47], which was sometimes related to inadequate facilities or the use of a patient’s relatives as interpreters [36]. Feeling unwelcome was cited in five studies [16, 23, 28, 31, 56]. Two studies cited the patient’s inability to choose which gender of providers they could see as a barrier to using services [39, 43]. The high workload of healthcare providers was identified as a barrier in three articles [16, 27, 31].

### Healthcare provider- patient communication

Seventeen studies reported language barriers in using SRH services [16, 20, 21, 27, 31–34, 36, 38, 40, 46, 47, 49, 53–55]. The language barrier was exacerbated by the lack of multicultural training in healthcare providers [19].

Five studies showed that communicating in many diverse languages promotes access to SRH services for migrant, internally displaced, asylum seeking and refugee women [20, 33, 38, 39, 47]. Similarly, three studies described how using a mobile app on smartphones to disseminate information about SRH services supports their use [37, 49, 50]. As well, providing a professional translator [27, 40, 47], using a family member as an interpreter [53, 57], and diversifying information sources [9] can break down communication barriers and thus promote the use of services.

### Financial accessibility

Seventeen studies reported financial factors as barriers to SRH services. These were primarily women’s low purchasing power [16, 22, 26, 29, 30, 43–45, 51], and the high cost of services [22–24, 33, 38, 40–42, 44, 48, 56, 57]. Financial barriers are particularly important in some settings where the cost of services is higher for migrant women than for native women [21]. This is also the issue when migrant women, unlike local women, do not have access to social security services [51].

### Knowledge of services

Twenty studies reported that inadequate knowledge limits women’s access to SRH services [15, 16, 18, 24, 25, 27–29, 33, 38, 40–42, 45–48, 53, 55, 56]. This lack of knowledge may relate to the availability or organization of services [25, 27, 28, 38, 45, 48, 55] or the presumed side effects of contraceptives [26, 27, 56]. In two studies, some women equated the use of contraceptives with abortion, revealing an obstacle to compliance with care [18, 45].

Practices that have been developed to increase migrant women’s knowledge and thus promote the use of reproductive health services were highlighted in two studies. This included the provision of health promotion representatives to improve migrant women’s knowledge about the availability of SRH services and thus encourage their use [38, 40].

### Cultural acceptability of services

Fifteen studies identified sociocultural considerations that conflict with the requirement for reproductive health services. Four studies found that some migrant, internally displaced, asylum seeking and refugee women have pro-natalist beliefs, which is conflicts with inherent need for contraceptive services [18, 20, 33, 51]. Nine articles describe the cultural imperative of sexual abstinence for unmarried women and for those whose husbands are away from home. This prevents women from using family planning or receiving STI or prenatal care [15, 17, 18, 20, 27, 28, 30, 42, 57]. In the same vein, one study found that the social imperative of virginity inhibited unmarried girls from obtaining cervical cancer screening, HPV vaccination, or family planning services [18]. In addition, three articles showed that the use of traditional medicine or other alternatives (e.g. self-medication) in the place of modern reproductive healthcare reduced the use of SRH services [22, 25, 36].

In one study, the fact that healthcare services did not allow an attendant for women in labor limited the use of labor and delivery services [54]. Similarly, the presence of religious symbols that are incompatible with migrant women’s beliefs hinder the use of healthcare centers [47]. Finally, women reported not accessing services due to experiencing feelings of shame when they were required to be examined naked during gynecological examinations, a necessity for prenatal consultations, STI management and family planning services [17].

In addition to sociocultural barriers, similar facilitating factors were reported. For example, six studies reported that programming of services by community groups [32], developing a partnership with community leaders [33, 47], and having home healthcare visits [27, 45] made it possible to remove cultural barriers to the use of SRH services. Similarly, a positive effect was seen in two studies when healthcare providers received training on person-centered care [31, 32]. Finally, four studies reported positive effects from the training of healthcare providers on cultural sensitivity and cultural communication [33, 38, 39, 47].

### Stigma/Discrimination

Five studies describe that the stigma of being a migrant, internally displaced person (IDP), asylum seeker or refugee was a barrier to the use of SRH services [17, 27, 32, 43, 49]. Feelings of discrimination were also reported in seven studies [21, 27, 28, 31, 38, 43, 54]. Finally, women were not motivated to seek preventive care as they considered it relatively less important in comparison to the multitude of other problems they faced [46].

### Autonomy in decision-making

Insufficient autonomy in decision-making was identified as a barrier to the use of SRH services. This reflects the low decision-making power of women, the effect of which was identified in nine studies [15, 16, 23, 25, 33, 35, 43, 55, 57]. Barriers related to decision-making autonomy are exacerbated by spouses’ low level of education [51] and lack of involvement in reproductive healthcare issues [57].

### Administrative factors

Four studies reported that the lack of documents required for healthcare access limits access to reproductive health services for migrant, internally displaced, asylum seeking and refugee women [21, 43, 44, 48].

## Discussion

This review provides an up-to-date synthesis of knowledge on the barriers and facilitating factors related to the use of SRH services by migrant, internally displaced, asylum seeking and refugee women. It thus offers insight into how to support the management of this vulnerable population regarding an important part of their healthcare needs. This synthesis discusses the geography of the studies, the populations studied, and the factors identified.

Geographically, Asia was the most represented continent, providing more than one-third of the articles reviewed (16 of 42). Studies based in countries from the African continent (n = 13), Europe (n = 10) and North America (n = 4) provided the remaining literature. Despite receiving important numbers of migrants, North America was relatively underrepresented in the literature retrieved. In 2020, the International Organization for Migration estimated that North America was the third region in the world -behind Europe and Asia- in terms of absolute numbers and proportions of migrants [1]. As well, few studies focused on West African countries. However, ongoing terrorism in this part of the world provokes massive internal displacements of populations, particularly in Nigeria, Mali, Niger, and Burkina Faso [58]. Future studies on access to SRH services should focus on the needs of women in West Africa.

Within the populations studied, internally displaced persons and refugees were relatively poorly represented (only 17 of 42 articles selected for this scoping review). However, the circumstances of their displacement, which is most often brutal, make them a particularly vulnerable sub-population that deserves to be better studied. Indeed, violent conflicts that lead to their rapid departure force refugees to abandon their belongings and property. Subsequently, most live in temporary settlement sites or camps, and they are at a particularly high risk of having their SRH rights violated [59]. Further research on access to reproductive healthcare services should focus exclusively on these forcibly displaced people.

The factors identified as barriers and facilitators were grouped into ten dimensions. These are geographic accessibility, availability of services, quality of services, communication, affordability, knowledge of services, cultural acceptability, stigma/discrimination, decision-making autonomy, and administrative factors. Overall, no factor emerged that was exclusively found to apply to migrants or IDPs or asylum-seekers or refugee women. In the studies that only focused on internally displaced women, language issues were not reported. This is understandable considering they are still within their country of origin.

With regards to the frequency of factors identified, lack of knowledge about services (n = 20), cultural unacceptability of services (n = 18), financial inaccessibility (n = 17), and language barriers (n = 17) were the main barriers to accessing to SRH services by migrant, internally displaced, asylum seeking and refugee women. Actions to improve access for this specific population should focus on these factors.

The most commonly faced barrier (from a frequency perspective) to accessing SRH services was insufficient knowledge about the services. These studies show that migrant, internally displaced, asylum seeking and refugee women are not aware of the services that are provided, nor are they aware when the healthcare service or clinics are open. These findings are in line with a scoping review that reported lack of knowledge and information was the main barrier to the use of reproductive health services for refugee girls [8]. The convergence of these results shows that among migrant, internally displaced, asylum seeking and refugee women, the lack of knowledge and information concerns not only girls but all ages of women. Displaced women undergo changes to their healthcare system, and thus, have lost the experiential capital they had accumulated in their land of origin. Language barriers contribute to this lack of knowledge about healthcare services. However, it emerged from the literature that the use of mobile applications on smartphones [37, 49, 50], having a variety of information sources [39], and the provision of health promotion representatives [38, 40] can break down language barriers and improve knowledge of migrant, internally displaced, asylum seeking and refugee women about SRH services.

This review also highlights cultural concerns that influence access to SRH services for migrant, internally displaced, asylum seeking and refugee women. For example, pro-natalist beliefs and traditions may not allow women to use family planning services. The same is true for social rejection of premarital sex and the social stigma of sex outside marriage. These long-acquired cultural beliefs are still very much alive in the migrant, internally displaced, asylum seeking and refugee women even when they move to and live in their host sites. So, healthcare providers for migrant, internally displaced, asylum seeking and refugee women must be trained in cultural sensitivity and cultural communication [33, 38, 39, 47], and in person-centered care [31, 32].

Although less frequently described, stigma and discrimination (n = 13) along with low decision-making autonomy (n = 9), are barriers that merit attention. To bring about significant improvement in access for migrant, internally displaced, asylum seeking and refugee women requires that women have the autonomy to make decisions. This is especially important because social stigma regarding reproductive health issues is prevalent in these communities.

Given the precariousness of their living conditions and the violence that has sometimes surrounded their displacement, the extent of mental health disorders could be particularly significant among migrant, internally displaced, asylum seeking and refugee women. These mental health disorders constitute a potential limit to access to SRH services, which has been little investigated. Taking mental health into account in future studies would enable a more complete understanding of the barriers to accessing SRH services for migrant, internally displaced, asylum seeking and refugee women.

### Strengths and weaknesses

A major strength of this review is that it has allowed for the development of a framework for analyzing the drivers and barriers to access to sexual and reproductive health services. This framework, an adaptation of Peters et al. [14], considers the lived experiences of the issues faced by migrant, internally displaced, asylum seeking and refugee women regarding sexual and reproductive healthcare access. It can be used as a framework for analyzing factors influencing the use of reproductive health services for these populations in future studies.

Another strength of this review is that we consulted a large number of databases (n = 8), which allowed for the retrieval and review of many articles. Similarly, the broad geographic scope allowed for the investigation of this issue in parts of the world that deserve further study. This synthesis provides an almost complete picture of the facilitating factors and barriers to the use of reproductive health services by migrant, internally displaced, asylum seeking and refugee women.

The main limitation of this study is the heterogeneity of the study population. Indeed, our study population includes people who were forced into displacement (IDPs, asylum seekers and refugees) and ordinary migrants whose displacement was planned. Thus, the challenges in accessing reproductive healthcare services may be different for each. Pooling these two subpopulations complicates the interpretation of the results. Future synthesis studies should focus on a more homogeneous population.

## Conclusion

Promoting access to sexual and reproductive health services, a fundamental human right, requires a good knowledge of the facilitating factors and obstacles to their access to such services. This scoping review provided an overview of the current literature on the subject. We identified ten groups of factors that promote or restrict access to reproductive healthcare services for migrant, internally displaced, asylum seeking and refugee women. Based on this evidence, we have built a conceptual framework that can be used for a holistic analysis of the barriers and facilitators of access to SRH services for migrant, internally displaced, asylum seeking and refugee women. Policymakers and health authorities must develop intervention strategies based on these factors to protect the reproductive healthcare rights of this specific population. The critical analysis of the literature also highlighted the need to take into account the mental health of migrant, internally displaced, asylum seeking and refugee women which, to date, has received little attention.

## Supporting information

S1 Appendix Search strategy applied in each database. (DOCX)
