# Correction: Barriers and facilitators of access to sexual and reproductive health services among migrant, internally displaced, asylum seeking and refugee women: A scoping review

**DOI:** 10.1371/journal.pone.0332052

**Published:** 2025-09-10

**Authors:** Pengdewendé Maurice Sawadogo, Drissa Sia, Yentéma Onadja, Idrissa Beogo, Gabriel Sangli, Nathalie Sawadogo, Assé Gnambani, Gaëtan Bassinga, Stephanie Robins, Eric Tchouaket Nguemeleu

The Funding statement for this article is incorrect. The correct Funding statement is as follows: This research was part of the SSRD project funded by IDRC-Canada.
