# Supplementary material for: Barriers and facilitators of access to sexual and reproductive health services among migrant, internally displaced, asylum seeking and refugee women: A scoping review
Source: PLoS One. 2023 Sep 14;18(9):e0291486. doi: 10.1371/journal.pone.0291486 (PMC10501608; doi:10.1371/journal.pone.0291486)
Supplement: S1 Appendix — (DOCX) [file pone.0291486.s001.docx]

**Appendix 1 : Search strategy applied in each database**

| Database | Search strategy | records |
| --- | --- | --- |
| Embase | (Refugees or Refugee or 'asylum seekers' or displaced or migrants or migrant or immigra* or emigration).ab,ti. OR exp refugee/ or exp asylum seeker/ or exp migrant/ OR exp migrant worker/ or exp migration/)  AND  (('Reproductive Health' or 'sexual Health' or 'Women Health' or 'Maternal health' or 'child health' or 'Adolescent Health' or 'family planning' or HIV or AID or STI or 'Sexual disease' or parturition or pregnancy or pregnancies or pregnant or gestat* or Prenatal or 'Pre natal' or Pre-natal or ante-natal or 'ante natal' or antenatal or 'peri natal' or perinatal or peri-natal or 'post natal' or post-natal or postnatal or vaccination or abortion or violence).ab,ti. OR exp reproductive health/ OR exp sexual health/ OR exp women's health/ OR exp maternal welfare/ OR exp adolescent health/ OR exp family planning/ OR exp sexually transmitted disease/ OR exp postnatal care/ OR exp prenatal care/ OR exp pregnancy/ OR exp sexually transmitted disease/ OR exp acquired immune deficiency syndrome/ OR exp vaccination/)  AND  ('Health Care' or Healthcare).ab,ti. OR exp health care/ OR accessibility OR utilization)  AND  (Barriers OR Obstacles OR Challenges OR Difficulties OR Issues OR Problems OR Facilitating factors OR facilitators) | 426 |
| MEDLINE | (Refugees or Refugee or 'asylum seekers' or displaced or migrants or migrant or immigra* or emigration).ab,ti. OR exp refugee/ or exp migrant/ )  AND  ('Reproductive Health' or 'sexual Health' or 'Women Health' or 'Maternal health' or 'child health' or 'Adolescent Health' or 'family planning' or HIV or AID or STI or 'Sexual disease' or parturition or pregnancy or pregnancies or pregnant or gestat* or Prenatal or 'Pre natal' or Pre-natal or ante-natal or 'ante natal' or antenatal or 'peri natal' or perinatal or peri-natal or 'post natal' or post-natal or postnatal or vaccination or abortion or violence).ab,ti. OR reproductive health/ OR sexual health/ OR women's health/ OR maternal welfare/ OR adolescent health/ OR Family Planning Services/ OR sexually transmitted disease/ OR postnatal care/ OR prenatal care/ OR pregnancy/ OR acquired immune deficiency syndrome/ OR vaccination/) AND '('Health Care' or Healthcare).ab,ti. OR health care/ OR (accessibility OR utilization).ab,ti.)  AND  (Barriers OR Obstacles OR Challenges OR Difficulties OR Issues OR Problems OR Facilitating factors OR facilitators).ab,ti. | 238 |
| CINAHL | (Refugees or Refugee or 'asylum seekers' or displaced or migrants or migrant or immigra* or emigration).ab,ti. OR (MH "Refugees+") OR (MM "Transients and Migrants")  AND  TI ( 'Reproductive Health' or 'sexual Health' or 'Women Health' or 'Maternal health' or 'child health' or 'Adolescent Health' or 'family planning' or HIV or AID or STI or 'Sexual disease' or parturition or pregnancy or pregnancies or pregnant or gestat* or Prenatal or 'Pre natal' or Pre-natal or ante-natal or 'ante natal' or antenatal or 'peri natal' or perinatal or peri-natal or 'post natal' or post-natal or postnatal or vaccination or abortion or violence ) OR AB ( 'Reproductive Health' or 'sexual Health' or 'Women Health' or 'Maternal health' or 'child health' or 'Adolescent Health' or 'family planning' or HIV or AID or STI or 'Sexual disease' or parturition or pregnancy or pregnancies or pregnant or gestat* or Prenatal or 'Pre natal' or Pre-natal or ante-natal or 'ante natal' or antenatal or 'peri natal' or perinatal or peri-natal or 'post natal' or post-natal or postnatal or vaccination or abortion or violence ) OR (MH "Reproductive Health") OR (MH "Sexual Health") OR (MH "Sexual Abuse+") OR (MH "Child Abuse, Sexual") OR (MH "Women's Health") OR (MH "Women's Health Services") OR (MH "Maternal Welfare") OR (MH "Maternal-Child Welfare") OR (MH "Adolescent Health") OR (MH "Adolescent Health Services") OR (MH "Family Planning+") OR (MH "Sexually Transmitted Diseases+") OR (MH "Postnatal Care+") OR (MH "Prenatal Care") OR (MH "Pregnancy+") OR (MH "Acquired Immunodeficiency Syndrome") OR (MH "Immunization-Vaccination Administration (Iowa NIC)")  AND  'TI ( 'Health Care' or Healthcare ) OR AB ( 'Health Care' or Healthcare ) OR (MH "Health Care Delivery, Integrated") OR (MH "Shared Services, Health Care") OR (MH "Health Care Delivery+") OR TI ( (accessibility OR utilization ) OR AB ( (accessibility OR utilization )  AND  TI ( Barriers OR Obstacles OR Challenges OR Difficulties OR Issues OR Problems OR Facilitating factors OR facilitators ) OR AB ( Barriers OR Obstacles OR Challenges OR Difficulties OR Issues OR Problems OR Facilitating factors OR facilitators ) | 245 |
| SCOPUS | ( refugees OR "asylum seekers" OR displaced OR migrant OR immigrant OR emigration ) AND ( healthcare OR "Reproductive Health" OR "sexual Health" OR "Women Health" OR "Maternal health" OR "child health" OR "Adolescent Health" OR 'family planning' OR HIV/AID OR STI OR 'Sexual disease' OR parturition OR pregnan* OR gestat* OR Prenatal OR 'Pre natal' OR Pre-natal OR ante-natal OR 'ante natal' OR antenatal OR perinatal OR 'post natal' OR post-natal OR postnatal OR vaccination OR abortion OR violence ) AND (Barriers OR Obstacles OR Challenges OR Difficulties OR Issues OR Problems OR factors OR facilitators) AND (accessib* OR utili* OR use) | 606 |
| Sciences Direct | (Refugees OR 'internal displaced') AND (Women OR child) AND (Barrier OR Facilitat) AND 'Healthcare accessibility' | 949 |
| Web of sciences | TOPIC: ((Refugees or Refugee or 'asylum seekers' or displaced or migrants or migrant or immigra* or emigration)) OR  TOPIC: (('Reproductive Health' or 'sexual Health' or 'Women Health' or 'Maternal health' or 'child health' or 'Adolescent Health' or 'family planning' or HIV or AID or STI or 'Sexual disease' or parturition or pregnancy or pregnancies or pregnant or gestat* or Prenatal or 'Pre natal' or Pre-natal or ante-natal or 'ante natal' or antenatal or 'peri natal' or perinatal or peri-natal or 'post natal' or post-natal or postnatal or vaccination or abortion or violence))  AND  TOPIC: (accessibility OR utilization)  AND  TOPIC: (Barriers OR Obstacles OR Challenges OR Difficulties OR Issues OR Problems OR Facilitating factors OR facilitators) | 671 |
| HINARI | (Refugees or Refugee or 'asylum seekers' or displaced or migrants or migrant or immigrant or emigration). All fields | 807 |
| COCHRANE LIBRARY | (Refugees or Refugee or 'asylum seekers' or displaced or migrants or migrant or immigra* or emigration):ti,ab,kw OR MeSH descriptor: [Refugees] explode all trees OR MeSH descriptor: [Refugees] explode all trees OR MeSH descriptor: [Transients and Migrants] explode all trees OR MeSH descriptor: [Transients and Migrants] explode all trees  AND  ('Reproductive Health' or 'sexual Health' or 'Women Health' or 'Maternal health' or 'child health' or 'Adolescent Health' or 'family planning' or HIV or AID or STI or 'Sexual disease' or parturition or pregnancy or pregnancies or pregnant or gestat* or Prenatal or 'Pre natal' or Pre-natal or ante-natal or 'ante natal' or antenatal or 'peri natal' or perinatal or peri-natal or 'post natal' or post-natal or postnatal or vaccination or abortion or violence):ti,ab,kw OR ('Health Care' or Healthcare):ti,ab,kw OR MeSH descriptor: [Reproductive Health] explode all trees OR MeSH descriptor: [Reproductive Health Services] explode all trees OR MeSH descriptor: [Sexual Health] explode all trees OR MeSH descriptor: [Maternal-Child Health Services] explode all trees OR MeSH descriptor: [Maternal-Child Health Services] explode all trees OR MeSH descriptor: [Family Planning Services] explode all trees OR MeSH descriptor: [Sexually Transmitted Diseases] explode all trees OR MeSH descriptor: [Postnatal Care] explode all trees OR MeSH descriptor: [Prenatal Care] explode all trees OR MeSH descriptor: [Pregnancy] explode all trees OR MeSH descriptor: [Acquired Immunodeficiency Syndrome] explode all trees OR MeSH descriptor: [Immunization Programs] explode all trees OR '('Health Care' or Healthcare):ti,ab,kw  AND  'MeSH descriptor: [Delivery of Health Care] explode all trees OR MeSH descriptor: [Health Services Accessibility] explode all trees OR (accessibility OR utilization):ti,ab,kw  AND  (Barriers OR Obstacles OR Challenges OR Difficulties OR Issues OR Problems OR Facilitating factors OR facilitators):ti,ab,kw | 272 |
| UNHCR website | "sexual and reproductive health" | 378 |
| OCHA Website | "sexual and reproductive health" | 108 |
| Open Grey | (migrants OR IDPs OR asylum seeker OR refugees) AND health | 22 |
